# Endocochlear potential contributes to hair cell death in TMPRSS3 hearing loss

**DOI:** 10.1172/JCI186395

**Published:** 2025-07-17

**Authors:** A. Eliot Shearer, Yuan-Siao Chen, Stephanie L. Rouse, Xiaohan Wang, Janmaris Marin Fermin, Kevin T.A. Booth, Jasmine Moawad, Nicole Bianca Libiran, Jinan Li, Hae-Young Kim, Michael Hoa, Rafal Olszewski, Jing-Yu Lei, Ernesto Cabrera, Douglas J. Totten, Bo Zhao, Jeffrey R. Holt, Rick F. Nelson

**Affiliations:** 1 Department of Otolaryngology and Communication Enhancement, Boston Children’s Hospital, Boston, Massachusetts, USA.; 2 Department of Otolaryngology Head and Neck Surgery, Harvard Medical School, Boston, Massachusetts, USA.; 3 Department of Otolaryngology-Head and Neck Surgery, and; 4 Department of Medical and Molecular Genetics, Indiana University School of Medicine, Indianapolis, Indiana, USA.; 5 Indiana University School of Medicine, Indiana University, Indianapolis, Indiana, USA.; 6 Auditory Development and Restoration Program, Neurotology Branch, National Institutes on Deafness and Other Communication Disorders, NIH, Bethesda, Maryland, USA.

**Keywords:** Cell biology, Otology, Drug therapy, Genetic diseases, Proteases

## Abstract

Pathogenic variants in the gene *TMPRSS3* are a common cause of hearing loss in humans, although the causal mechanisms remain unknown. Previous work has shown that *Tmprss3^Y260X/Y260X^* mice exhibit normal hair cell development, mechanosensory transduction, and spiral ganglion patterning, but experience rapid hair cell death from P12 to P14 at the onset of hearing. Here, we demonstrate that *Tmprss3^Y260X/Y260X^* mice display an early and temporary spike in endocochlear potential (EP) prior to the onset of hair cell death. In vitro experiments with cochlear explants from *Tmprss3^Y260X/Y260X^* mice and in vivo studies with *Tmprss3^Y260X/Y260X^* mice crossed with 2 different mutant models that lacked EP generation promoted hair cell survival. Furthermore, systemic administration of furosemide, a drug that reduces EP in vivo, reduced hair cell death in *Tmprss3^Y260X/Y260X^* mice. These findings suggest that extracellular factors, including EP, play a role in TMPRSS3-related hair cell survival and hearing loss, and suggest that modulating EP could be a therapeutic strategy.

## Abstract 2

Shearer et al. reveal that high intracochlear electrical potential contributes to hair cell degeneration and is a therapeutic target for TMPRSS3-hearing loss

## Graphical abstract

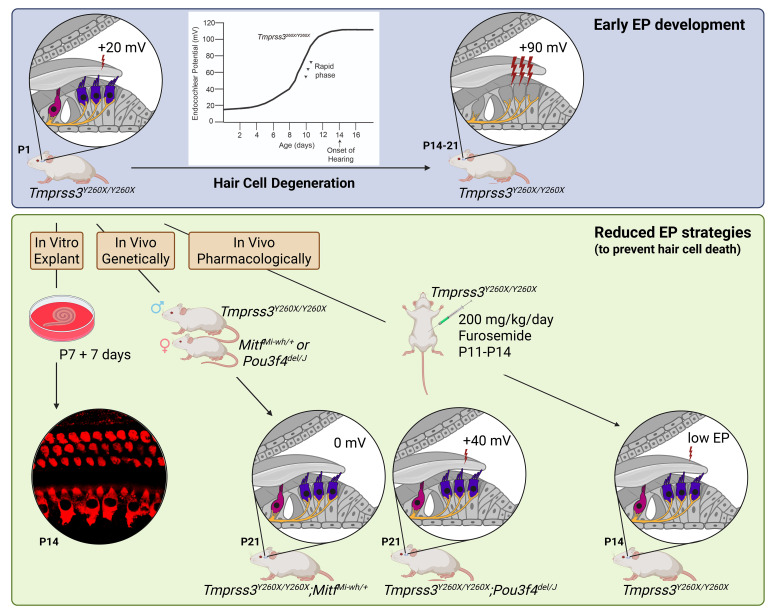

## Introduction

For the majority of genes associated with hearing loss (HL), the underlying pathologic mechanism is not known. Determining the precise molecular mechanism underlying each form of genetic HL is critical for development of new treatment modalities, which currently are limited to hearing aids and cochlear implants but in the near future may include correction of underlying pathology with molecular or gene therapies. One gene for which the underlying disease mechanism is still elusive is *TMPRSS3*.

Biallelic pathogenic variants in *TMPRSS3* cause 2 forms of sensorineural HL: early-onset severe to profound HL (DFNB10) and postlingual, progressive, high-frequency HL (DFNB8) (1). *TMPRSS3* is commonly implicated as a cause of human HL (2). We and others have previously shown that, unlike other forms of genetic HL, *TMPRSS3*-related HL is associated with variable speech perception outcomes after cochlear implantation in adults and is correlated with duration of deafness, possibly due to reduced spiral ganglion neuron (SGN) function (3–5). In contrast, in children with TMPRSS3-related HL, cochlear implant outcomes are more positive (6). Given its relatively substantial contribution to genetic HL and the variable cochlear implant outcomes associated with *TMPRSS3*-related HL, determination of the function of *TMPRSS3* and how its dysfunction results in HL is critical for a comprehensive understanding of DFNB8/10.

The *TMPRSS3* gene encodes the transmembrane serine protease III protein, a widely expressed protein with multiple tissue-specific isoforms (7). To date, there are more than 70 reported pathogenic HL-associated variants in *TMPRSS3* (8, 9). In the inner ear, *TMPRSS3* is expressed in inner hair cells (IHCs), outer hair cells (OHCs), and in type II, but not type I, SGNs (7, 10, 11). The previously developed *Tmprss3*-null mouse model (*Tmprss3^Y260X/Y260X^*) shows rapid degeneration of cochlear hair cells (HCs) beginning at the onset of hearing at P12, leading to compete loss of HCs by P14 and resulting in profound deafness (7, 11). HCs s develop normally, but they quickly undergo cell death at the onset of hearing, a unique pathology compared with other forms of genetic HL.

The mechanism for HC death at the onset of hearing in *Tmprss3^Y260X/Y260X^* mice is not understood. However, due to the coincident timing of normal development of the endocochlear potential (EP) (12) and rapid HC degeneration in *Tmprss3^Y260X/Y260X^* mice, we hypothesized that EP may play a role in HC loss and subsequent deafness in *Tmprss3^Y260X/Y260X^* mice. The apical surface of cochlear HCs is bathed in endolymph, which is high in extracellular K^+^ and provides the +90 mV EP. Interestingly, the period at which massive HC death occurs in *Tmprss3^Y260X/Y260X^* mice coincides with development of the EP, because there is a rapid increase from +16 mV to +60 mV, reaching the mature +90 mV by P15 (12). The EP is generated by the cells of the stria vascularis and provides a steep electrochemical gradient that drives sensory transduction current into HCs, carried primarily by K^+^ and Ca^2+^ (13). One route for K^+^ efflux from HCs is via big potassium (BK) channels, which are large-conductance, calcium-activated K^+^ channels. *KCNMA1* is the gene that produces the pore-forming α subunit of BK channels, which localize on the neck of mature IHCs (11, 14). Normally, *Kcnma1* is first expressed at P12; however, KCNMA1 channels are not detected in IHCs of *Tmprss3^Y260X/Y260X^* mice (11, 15). Interestingly, KCNMA1-null mice have normal hearing at 4 weeks of age and develop a mild, progressive, high-frequency HL based on alterations of OHC function (16). Based upon the temporal correlation between rapid HC death with EP maturation, along with alterations in HC K^+^ channel expression, we hypothesize that alterations of EP contribute to HC death and HL in *Tmprss3^Y260X/Y260X^* mice.

Here, we investigated this hypothesis by modifying the intracochlear environment and assessing effects on HC survival in *Tmprss3^Y260X/Y260X^* mice both in vitro, using a cochlear explant model, and in vivo, crossing *Tmprss3^Y260X/Y260X^* mice with 2 mouse strains that lack development of EP. We also measured EP directly in *Tmprss3^Y260X/Y260X^* mice and show that EP aberration is associated with the rapid HC death and HL in these mice. In addition, pharmacologic reduction of the EP with systemic administration of furosemide led to reduction in HC death, providing a new possible avenue for treatment of this common cause of human HL.

## Results

### HCs show normal early development and function in Tmprss3^Y260X/Y260X^ mice.

**Figure 1:**
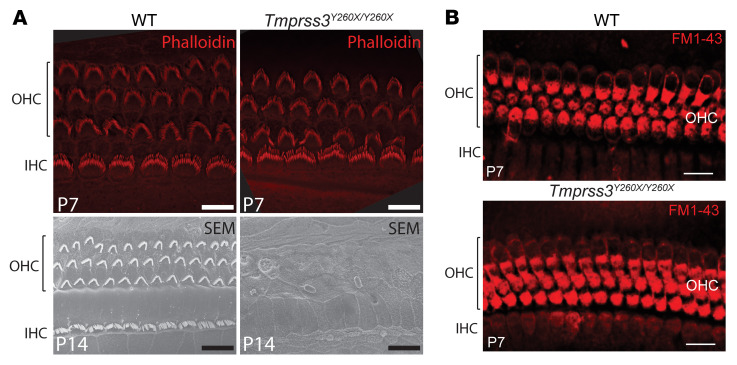
*Tmprss3^Y260X/Y260X^* mice demonstrate normal HC and stereocilia morphology and physiology prior to rapid-onset degeneration. (**A**) Phalloidin immunostaining and scanning electron microscopy (SEM) show normal architecture of IHCs and OHCs at P6 and P14 in wild-type mice and complete HC degeneration at P14 in *Tmprss3^Y260X/Y260X^* mice. Scale bars: 10 μm (P7), 20 μm (P14). (**B**) FM1-43 uptake by HCs in *Tmprss3^Y260X/Y260X^* mice is equivalent to that of wild-type mice at P7. Scale bars: 20 μm.

We evaluated the morphology and function of HCs in early stages of postnatal development in *Tmprss3^Y260X/Y260X^* mice prior to the onset of hearing and before the previously identified rapid and complete HC degeneration of IHCs and OHCs from P12 to P14. At P6, the HCs showed typical stereocilia morphology and cellular arrangement via phalloidin immunostaining (Figure 1A). The hair bundle ultrastructure of *Tmprss3^Y260X/Y260X^* mice is indistinguishable from that of heterozygous and wild-type littermates. Mechanosensory transduction currents in mouse HCs are detected at P0 at the base and reach mature levels between P5 and P10 in a tonotopic gradient from base to apex (17, 18). We noted typical HC uptake of FM1-43, a styryl dye that specifically labels HCs via uptake through functional sensory transduction channels, at P7 (Figure 1B). Consistent with prior reports, we found that *Tmprss3^Y260X/Y260X^* IHCs and OHCs undergo rapid and complete degeneration by P14 (Figure 1A). These data indicate normal early development and function of HCs in *Tmprss3^Y260X/Y260X^* mice prior to rapid degeneration.

### SGN patterning is normal at early stages in Tmprss3^Y260X/Y260X^ mice.

**Figure 2:**
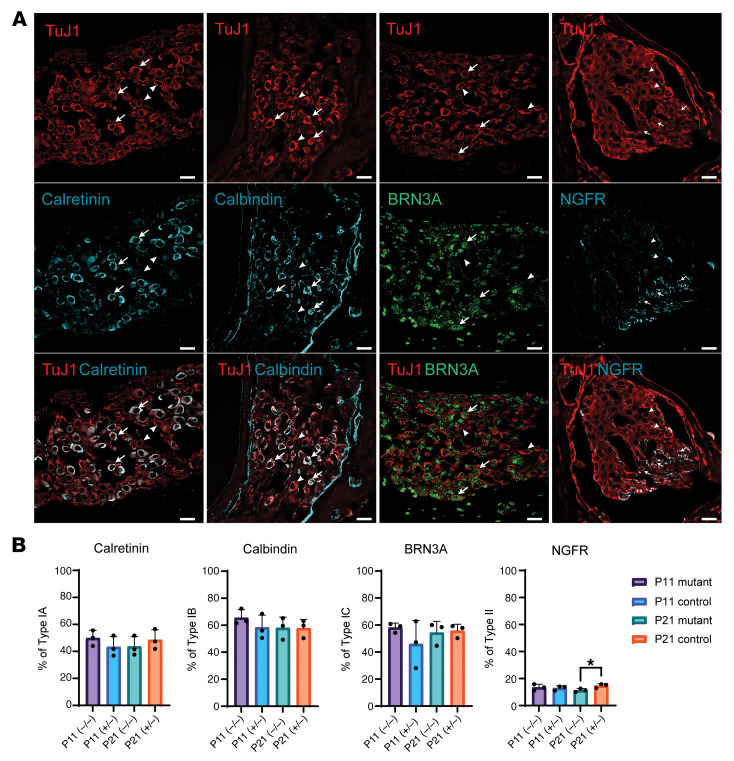
Spiral ganglion subtype composition is normal in *Tmprss3^Y260X/Y260X^* mice, aside from type II SGN increase. (**A**) Representative sections of spiral ganglion taken at P11 stained for neuron-specific class III β-tubulin (TuJ1) and antibodies specific for type IA SGNs (calretinin), type IB SGNs (calbindin), type IC SGNs (POU4F1, BRN3A), and type II SGNs (NGFR). Top row shows TuJ1 channel, middle row shows SGN subtype-specific channel, and the bottom row shows merged channels. Arrows indicate costaining; arrowheads indicate negative costaining. Scale bars: 20 μm. (**B**) Counted subtype-specific neurons at P11 and P21 for *Tmprss3^Y260X/Y260X^* and wild-type mice show no difference aside from increased type II SGNs in wild-type mice at P21. Each point represents cell count from 1 cochlea (*n* = 3 cochleae per genotype and day).

SGNs innervate IHCs and OHCs and consist of type I and type II SGNs. Type I SGNs compose 95% of SGNs, transmit sound information from the IHCs to the central nervous system, and consist of 3 unique subtypes (IA, IB, and IC) based upon single cell transcriptomic profiling (10, 19) and spontaneous firing rates (20). Type II SGNs make up the remaining 5% of the SGN population, synapse on multiple OHCs, and are not essential for sound transmission; instead, they are likely involved in damage perception (21). SGNs undergo major refinement in molecular phenotype in the first few postnatal weeks (19). Proper segregation of type I and type II SGNs and subtype specification of type I SGNs is dependent upon the spontaneous activity of HCs and functional sensory transduction prior to the onset of hearing (19). Genetically engineered mice with defective synaptic transmission or sensory transduction have severe alterations in the type 1 SGN subtyping (19). TMPRSS3 expression is not detected in type I SGNs and is limited to only type II SGNs (10). To test if loss of TMPRSS3 affected SGN function, we profiled the composition of SGNs in *Tmprss3^Y260X/Y260X^* mice at early time points (Figure 2). The total number of Tuj1-positive SGNs was no different between *Tmprss3^Y260X/Y260X^* and control mice (Supplemental Figure 1; supplemental material available online with this article; https://doi.org/10.1172/JCI186395DS1). Staining with antibodies specific for type IA (calretinin), type IB (calbindin), type IC (BRN3A), and type II (nerve growth factor receptor [NGFR] SGNs revealed identical subtype composition between the control mice (*Tmprss3^Y260X/+^*) and *Tmprss3^Y260X/Y260X^* mice at P11 (Figure 2, A and B). Despite HC degeneration by P14 in *Tmprss3^Y260X/Y260X^* mice, no significant differences in type I SGNs were observed at P21. The type II SGNs were slightly, though significantly, decreased in *Tmprss3^Y260X/Y260X^* mice at P21. These data demonstrate that loss of expression of TMPRSS3 does not affect activity-dependent subtype patterning of type 1 SGNs and provides further support for the indication that loss of TMPRSS3 does not disrupt HC transduction, which is critical to IHC development.

### HC death in Tmprss3^Y260X/Y260X^ mice is mediated by the intracochlear environment.

The endolymph within the scala media comprises extracellular fluid containing 157 mM K^+^ that is dependent on the KCNJ10 channel of the lateral wall of the cochlea (22). The positive EP within the scala media of 80–100 mV is generated by the stria vascularis and reaches peak levels at P14 at the onset of hearing (23).

**Figure 3:**
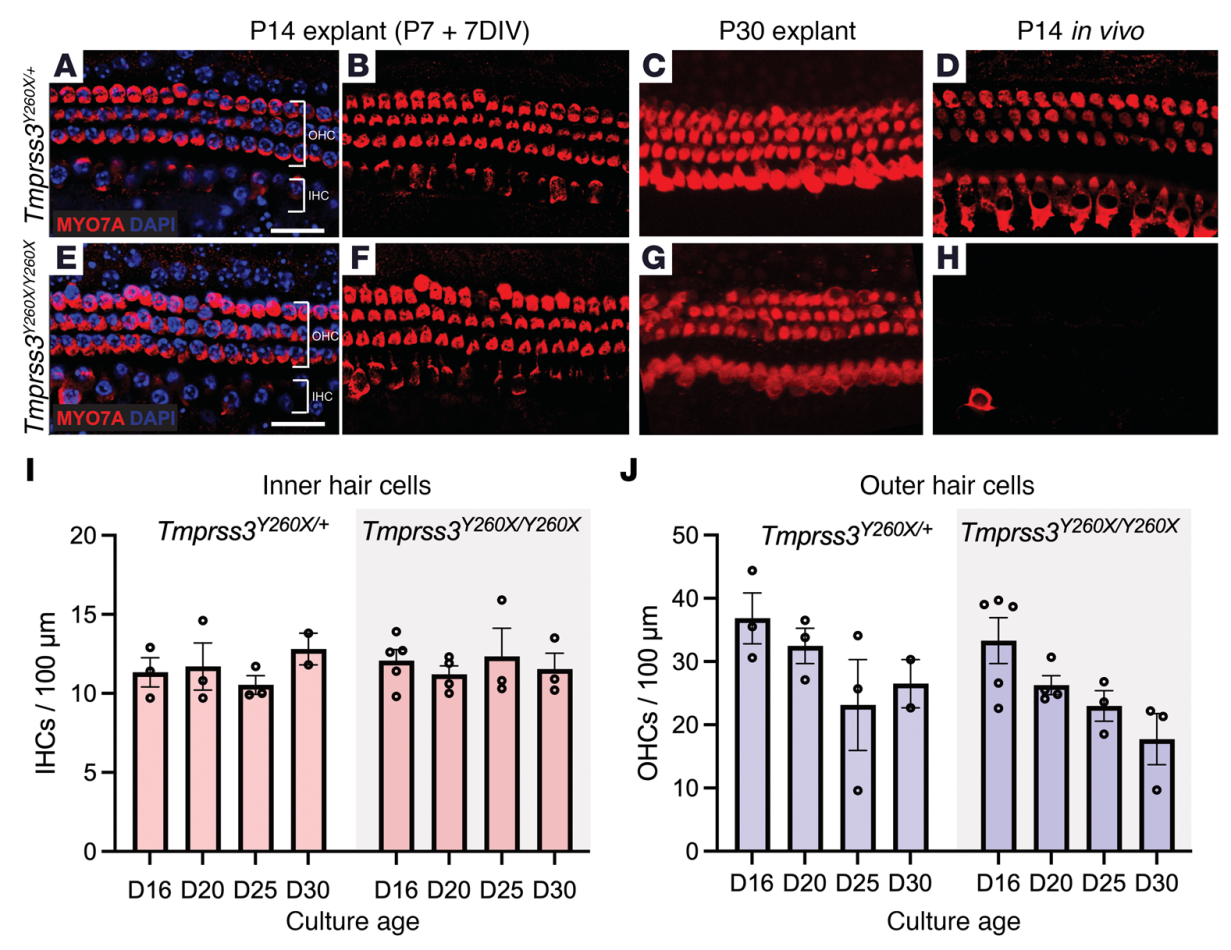
Cochlear explant cultures implicate intracochlear environment in HC death in *Tmprss3^Y260X/Y260X^* mice. Representative images of cochlear explant cultures from control (*Tmprss3^Y260X/+^*) (**A** and **B**) and *Tmprss3^Y260X/Y260X^* (**E** and **F**) mice. *Tmprss3^Y260X/Y260X^* explant at P14 equivalent (P7 explant + 7 days in vitro [DIV]) demonstrates no HC degeneration of either IHCs or OHCs, which is maintained to P30 explant (**C** and **G**). *Tmprss3^Y260X/Y260X^* mice display near complete HC degeneration at P14 in vivo (**D** and **H**). Scale bar: 20 μm. (**I** and **J**) Quantification of IHCs and OHCs in explant culture for control and *Tmprss3^Y260X/Y260X^* mice demonstrating slowly progressive OHC loss in both. There was no significant difference in cell counts up to 30 DIV (D30) (*P* > 0.11 in all cases). Each point represents the cell count from 1 culture. Exact Wilcoxon rank-sum test (Mann-Whitney *U* test) was used to compare 2 groups (*Tmprss3^Y260X/+^* and *Tmprss3^Y260X/Y260X^*) in each culture age. Up to 5 cultures were quantified for each day, but only 2 cultures were available for D30 control (see Supporting Data Values for full details).

We had previously shown that inner ear organoids derived from stem cells of *Tmprss3^Y260X/Y260X^* mice are morphologically and functionally identical to wild-type inner ear organoids and do not undergo the same rapid degeneration seen in vivo (24). Based on prior observations of sudden IHC and OHC death in *Tmprss3^Y260X/Y260X^* mice between P12 and P14 (11, 15), we hypothesized that the extracellular environment — the EP, in particular — may contribute to the early rapid HC death in *Tmprss3^Y260X/Y260X^* mice. To test this hypothesis and investigate if altering the extracellular environment could prevent HC death, we performed cochlear explant cultures. Mouse cochleae were microdissected at P7 and the sensory epithelium was placed in explant culture media for an additional 7 days in vitro (DIV). We observed complete preservation of HCs in the *Tmprss3^Y260X/Y260X^* mice (Figure 3).

To determine the length of survival in culture, we cultured P7 *Tmprss3^Y260X/Y260X^* explants for up to 30 days (Figure 3, A–I). Although we observed some loss of OHCs with progressively longer culture times, there was no statistically significant difference in HC survival between *Tmprss3^Y260X/Y260X^* explants and control *Tmprss3^Y260X/+^* explants (Figure 3J; *n* = 3–5 cultures per condition). This supported our hypothesis that the extracochlear environment causes massive HC death in *Tmprss3^Y260X/Y260X^* mice.

### EP measurement in mouse models.

**Figure 4:**
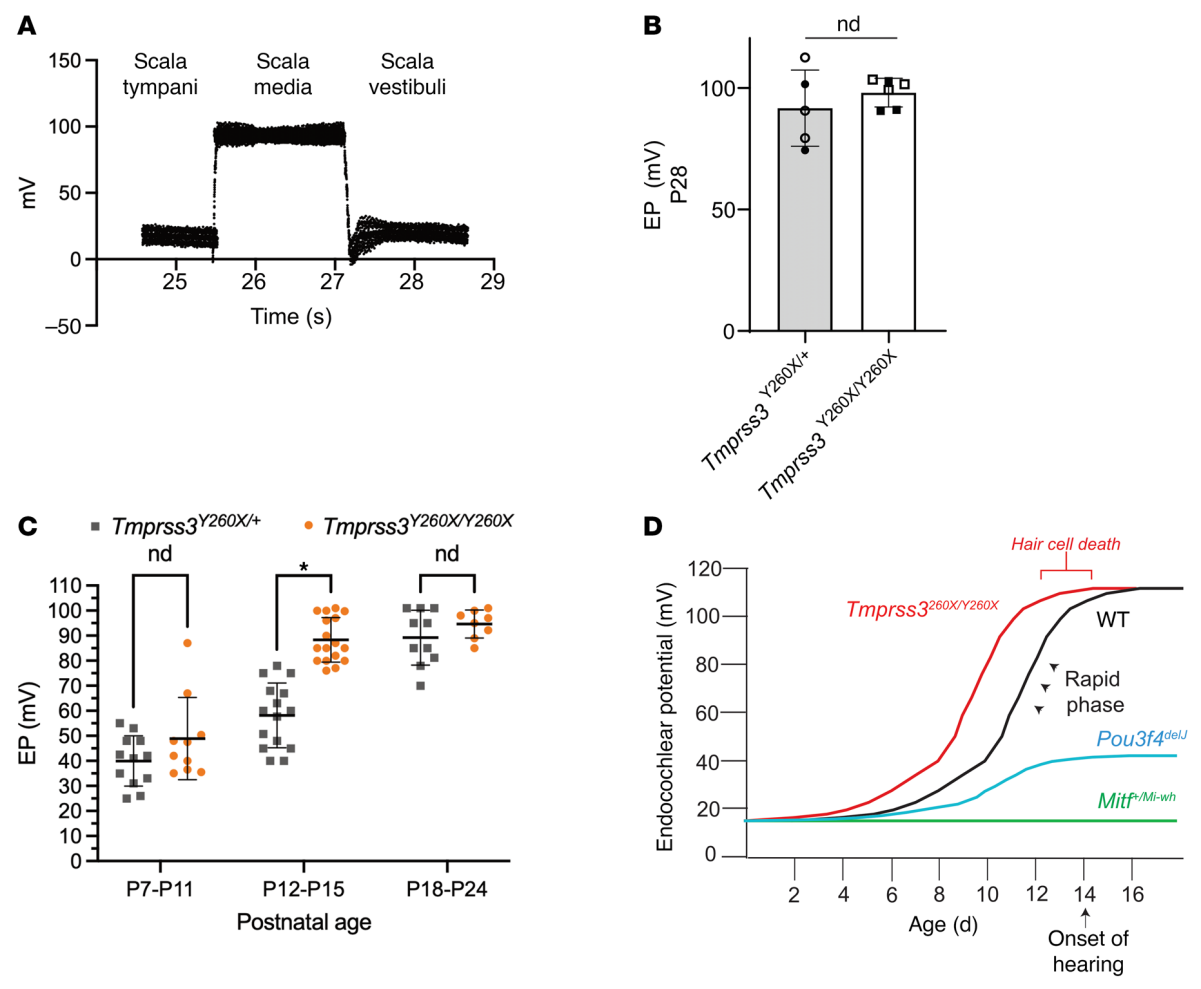
Direct EP measurements show supraphysiologic rise in EP in *Tmprss3^Y260X/Y260X^* mice. (**A**) EP was recorded through the basal turn of left cochleae in live mice ranging from P7 to P24. Representative tracing of EP recorded from the scala media of a live control mouse. (**B**) Direct EP recordings from P28 mice demonstrating no difference between control (*n* = 5) (*Tmprss3^+/–^*) and *Tmprss3^Y260X/Y260X^* (*n* = 6) mice. Open circles and squares represent female mice. Filled circles and squares represent male mice. nd, no statistical difference determined. (**C**) Direct EP recordings from mouse models. Each data point represents 1 reading from 1 animal and is plotted as the mean with SD. Mean EP at P12–P15 is significantly different (*P* < 0.0001) between *Tmprss3^Y260X/Y260X^* and control (*Tmprss3^Y260X/+^*) mice. Wilcoxon rank-sum test (Mann-Whitney *U* test) was used to compare 2 groups (control *Tmprss3^Y260X/+^* and experimental *Tmprss3^Y260X/Y260X^*) in each postnatal age group (P7–11: *n* = 12, *n* = 11; P12–15: *n* = 15, *n* = 11; P18–P24: *n* = 10, *n* = 9, for control and experimental mice). (**D**) Schematic demonstrating time frame of HC death in *Tmprss3^Y260X/Y260X^* mice in the context of physiologic rapid phase of EP development. Neither *Pou3f4^delJ^* nor *Mitf^Mi-wh/+^* knockout mice generated EP and neither had HC degeneration despite profound HL.

*Tmprss3* transcript expression is detected in the rare spindle root cells of the lateral wall and not within the major stria vascularis cells such as the marginal, intermediate, or basal cells (25). To determine if TMPRSS3 expression alters EP, we first performed EP measurement on adult mice at P28. We observed no difference in mature EP between *Tmprss3^Y260X/Y260X^* and control mice regardless of sex (Figure 4, A and B).

Next, we sought to evaluate EP dynamics in *Tmprss3^Y260X/Y260X^* mice during the critical period of EP development from P7 to P24. Similar to our adult measurements, we directly measured EP in live control and *Tmprss3^Y260X/Y260X^* mice, using glass micropipettes inserted into the scala media. The EP was recorded as the voltage difference between the micropipette’s stable positions in the scala media and the scala tympani (Figure 4A).

We found that the EP was, on average, 8.9 mV higher at ages P7–P11 in *Tmprss3^Y260X/Y260X^* mice compared with *Tmprss3^Y260X/+^* mice (48.9 ± 16.4 mV vs. 40.0 ± 10.0 mV, respectively) (Figure 4C). This difference was not statistically different (*P* = 0.26), as measured by a Wilcoxon rank-sum test (Mann-Whitney *U* test). The EP was 30 mV higher in *Tmprss3^Y260X/Y260X^* mice than in controls when tested at P12–P15 (88.3 ± 8.9 mV vs. 58.2 ± 12.9 mV, respectively; *P* < 0.0001) (Figure 4C).

After the onset of hearing, the EP stabilized at approximately 90–95 mV at P18–P21 and was not significantly different between *Tmprss3^Y260X/Y260X^* and control mice (89.2 ± 10.9 mV vs. 94.7 ± 5.6 mV, respectively). Thus, the premature rise in EP noted here in the *Tmprss3^Y260X/Y260X^* mice prior to P16 occurred concurrently with previously identified complete HC degeneration. The rapid increase in EP from P10 through the onset of hearing at P14 is temporally correlated with the rapid HC degeneration between P12 and P14 seen in *Tmprss3^Y260X/Y260X^* mice (Figure 4D). This early increase in EP may be a secondary consequence of an as-yet unidentified role of TMPRSS3 in the lateral wall, the lack of KCNMA1 expression in the *Tmprss3^Y260X/Y260X^* HCs, or a consequence of HC degeneration.

### Reduction of EP in vivo prevents HC death.

To further investigate the role of the EP on TMPRSS3-mediated HC death in vivo, we crossed the *Tmprss3^Y260X/Y260X^* line with the 2 different mutant mouse lines: Microphthalmia-White (*Mitf*^+/Mi-wh^) and *Pou3f4^delJ^*. *Mitf*^+/Mi-wh^ mice are a model for human deafness-pigmentation syndromes, Waardenburg syndrome type 2a, and Tietz syndrome, caused by mutations in *MITF* gene and characterized by profound deafness along with melanocyte deficiency. *Mitf*^+/Mi-wh^ mice carry heterozygous mutations in the *Mitf* gene and have profound HL and an EP that is less than 20 mV in adult mice (26, 27). Progressive OHC loss is observed in the *Mitf*^+/Mi-wh^ mice by P28 in a tonotopic gradient from base to apex (26). *Pou3f4^delJ^* mice are a model for human X-linked nonsyndromic deafness (DFN3) caused by mutations in the gene *POU3F4*, which is a transcription factor. Compared with controls, *Pou3f4^delJ^* mice have reduced EP (85 mV vs. 38 mV, respectively) (28). POU3F4 is required for generation of the EP but not for generation of cochlear HCs (28). As anticipated, we confirmed a lack of EP in the *Tmprss3^Y260X/+^;Mitf^+/Mi-wh^* mice at 1 month of age (*n* = 3; EP = 0 mV). Unsurprisingly, due to a complete lack of EP, these mice had profound HL as measured by auditory brainstem response (ABR) at all frequencies tested at P16 (Supplemental Figure 2).

**Figure 5:**
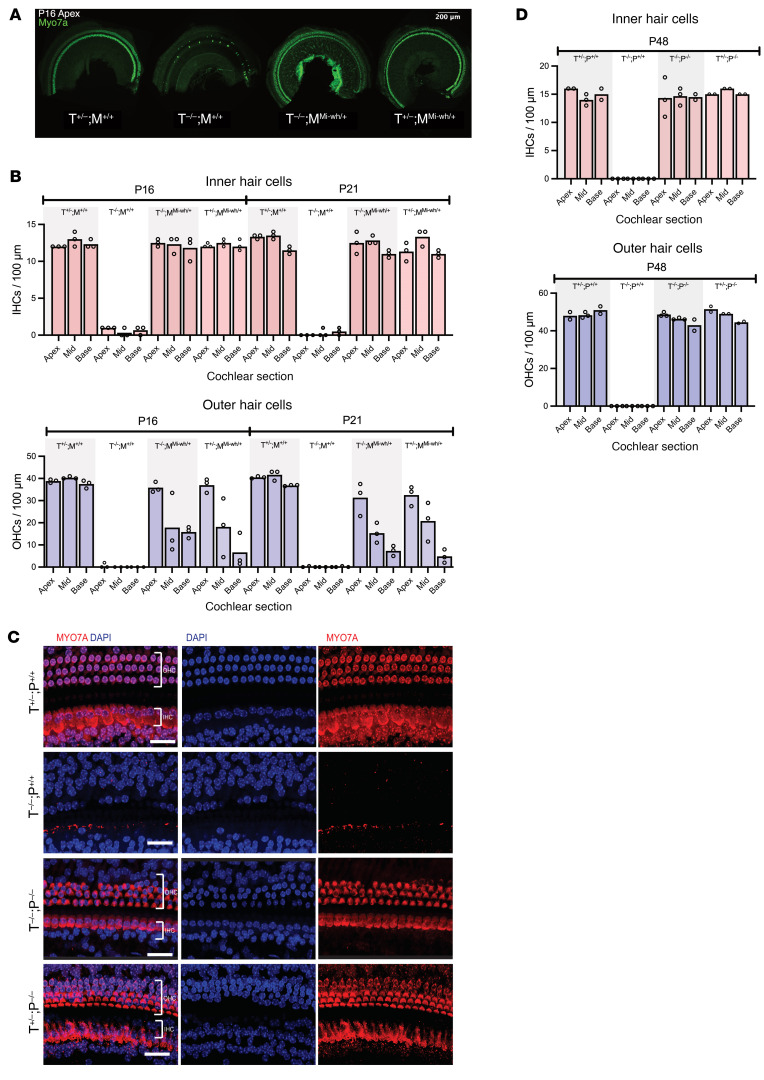
Rescue of cochlear HCs in *Tmprss3^Y260X/Y260X^* mice through reduction of EP via crossing *Mitf^Mi-wh/+^* mice and *Pou3f4^delJ^* mice. (**A**) Representative cochlear sections of *Tmprss3^Y260X/Y260X^* (T^–/–^) mice crossed with *Mitf^Mi-wh/+^* (M^Mi-wh/+^) mice. Sections shown are cochlear apex at P16, labelled with Myo7a. (**B**) IHC and OHC counts per 100 μm at P16 and P21 for T^–/–^ and M^Mi-wh/+^ crosses. Each dot represents the average of both ears for the same mouse; *n* = 3 mice per condition. Note loss of OHCs at P16 and P21 in M^Mi-wh/+^ mouse lines. (**C**) Representative cochlear sections of *Pou3f4^delJ^* (P^–/–^) crosses at P48. Cross of P^–/–^ with T^–/–^ mice rescues HCs. Scale bar: 30 μm. Staining is with Myo7a (red) and phalloidin (blue). (**D**) IHC and OHC counts for per 100 μm at P48 for *Pou3f4^delJ^* (P^–/–^) crosses. There is no loss of OHCs noted over time in this model. *n* = 2 or 3 mice per condition.

Next, we examined the loss of EP on HC survival and, intriguingly, there was a dramatic preservation of both IHCs and OHCs in the *Tmprss3^Y260X/Y260X^;Mitf^+/Mi-wh^* double-mutant mice (Figure 5, A and B). Although there was robust preservation of IHCs in *Tmprss3^Y260X/Y260X^;Mitf^+/Mi-wh^* mice (Figure 5B), we observed a base-to-apex gradient of OHC loss in *Tmprss3^Y260X/Y260X^;Mitf^+/Mi-wh^* mice that was not significantly different than *Tmprss3^Y260X/+^;Mitf^+/Mi-wh^* mice and is consistent with previously reported HC loss in the mouse line *Mitf^+/Mi-wh^* (Figure 5B) (29). We also observed no significant progression in OHC cell death P16 to P21 (Figure 5B).

To confirm this finding in an additional model that does not exhibit progressive HC loss, we generated *Tmprss3^Y260X/Y260X^;Pou3f4^delJ/Y^* (male) or *Tmprss3^Y260X/Y260X^;Pou3f4^delJ/delJ^* (female) double-mutant mice. Analysis of P48 mice revealed complete preservation of both IHCs and OHCs regardless of sex (Figure 5, C and D). Despite complete HC preservation, ABR testing of adult (P28) double-mutant mice (*Tmprss3^Y260X/Y260X^;Pou3f4^delJ^*) demonstrated profound HL, like we observe in *Tmprss3^Y260X/Y260X^* and in *Pou3f4^delJ^* mutant mice (Supplemental Figure 3). This hearing result is expected given the suspected low EP in double-mutant mice (*Tmprss3^Y260X/Y260X^;Pou3f4^delJ^*). Taken together, these data confirm that EP in vivo plays a major role in the pathogenesis of HC death in *Tmprss3^Y260X/Y260X^* mice.

### KCNMA1 channels are not expressed in surviving HCs.

**Figure 6:**
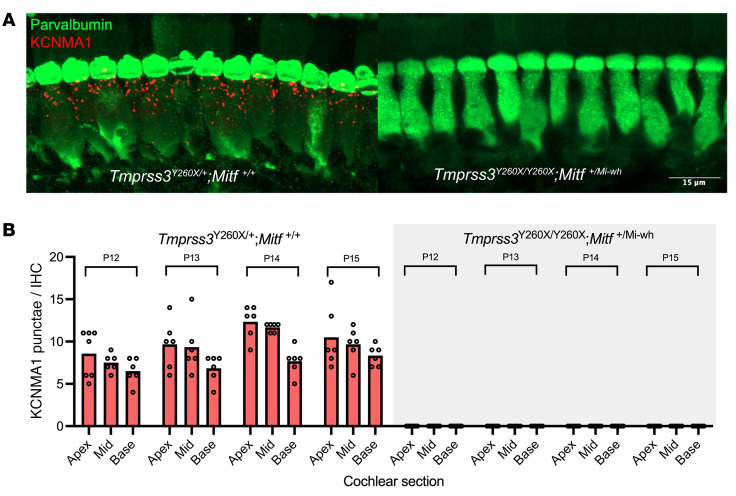
Reduced EP does not rescue KCNMA1 expression. (**A**) Representative sections from the base of P15 cochleae from control (*Tmprss3^Y260X+-^;Mitf^+/+^*) and double-mutant mice (*Tmprss3^Y260X/Y260X^; Mitf^Mi-wh/+^*) demonstrate lack of expression of the outward-rectifying K^+^-channel KCNMA1 surviving HCs that lack *Tmprss3* expression. (**B**) Counts of KCNMA1 puncta show a base to apex gradient of expression in control mice at P12 (*n* = 9), P13 (*n* = 9), P14 (*n* = 6), and P15 (*n* = 7) and lack of KCNMA1 expression in *Tmprss3^Y260X/Y260X^ Mitf^Mi-wh/+^* mice at P12 (*n* = 9), P13 (*n* = 6), P14 (*n* = 7), and P15 (*n* = 6). Note there were no punctae for *Tmprss3^Y260X/Y260X^ Mitf^Mi-wh/+^* mice (all values were 0).

Prior proteomic analysis of cochlea from control and *Tmprss3^Y260X/Y260X^* mice revealed reduced expression of APOA1, a KCNMA1-associated protein (11). Immunohistochemical staining demonstrated complete absence of KCNMA1 puncta near the apex of *Tmprss3^Y260X/Y260X^* HCs at P12 prior to degeneration (11). To determine if HC survival after P14 restored expression of KCMA1, we performed antibody labelling in *Tmprss3^Y260X/Y260X^;Mitf^+/Mi-wh^* mice. In control mice (*Tmprss3^Y260X/+^;Mitf^+/+^*), KCNMA1 expression increased from P12 to P15 in the previously reported base-apex gradient (14) (Figure 6, A and B). Conversely, although IHCs and OHCs survive due to the reduced EP in the *Tmprss3^Y260X/+^;Mitf^+/Mi-wh^* model (described earlier in *Reduction of EP in vivo prevents HC death*), there is complete lack of expression of KCNMA1 in the *Tmprss3^Y260X/Y260X^;Mitf^+/Mi-wh^* mice at P15 (Figure 6, A and B). Therefore, although reduction in EP results in HC survival in *Tmprss3^Y260X/Y260X^* mice, expression of KCNMA1 requires expression of functional TMPRSS3.

### Pharmacologic reduction in EP rescues HCs in Tmprss3^Y260X/Y260X^ mice.

**Figure 7:**
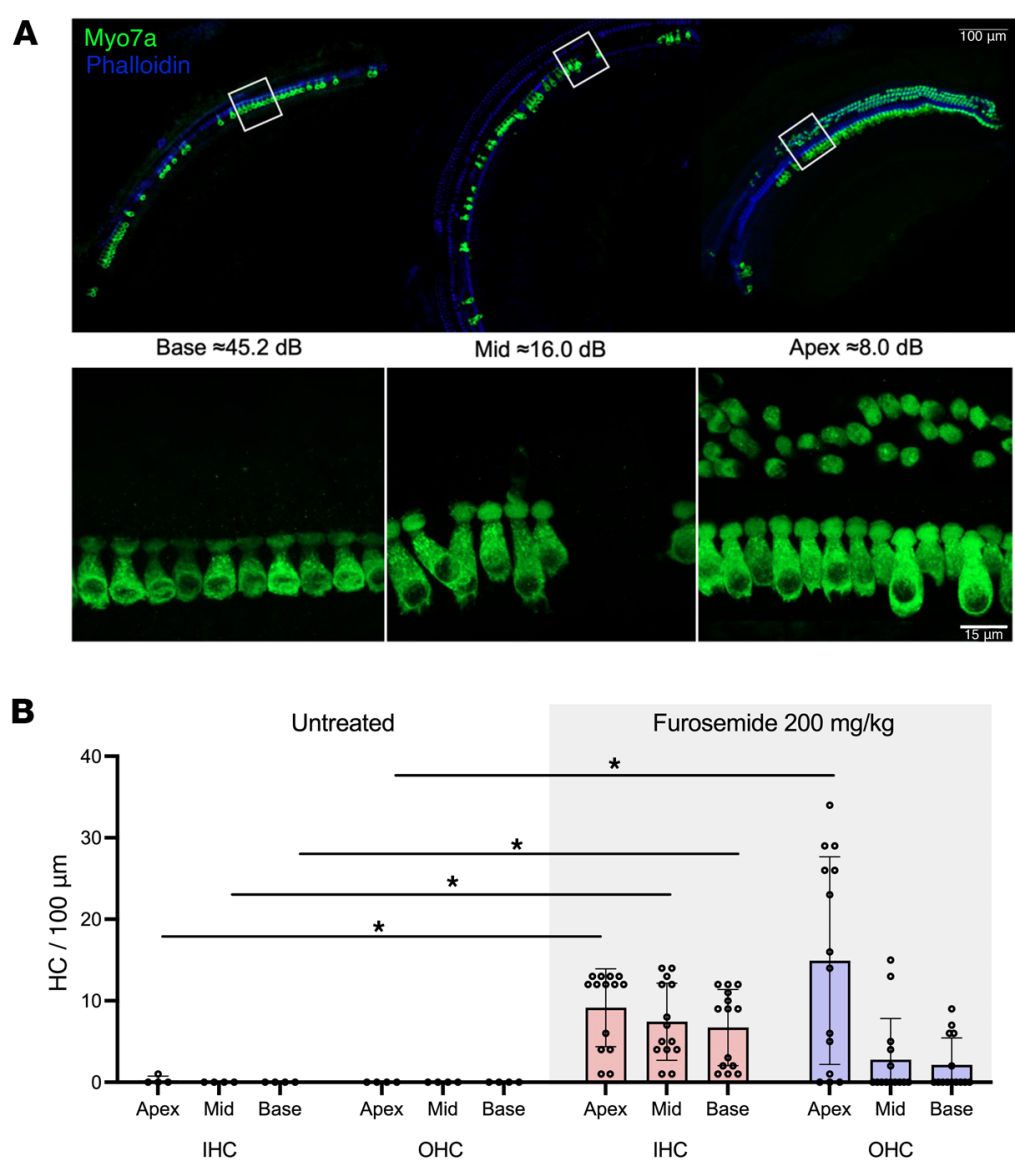
Pharmacologic reduction of EP using furosemide rescues HCs. (**A**) Administration of 200 mg/kg furosemide once daily from P10 to P14 leads to HC survival in *Tmprss3^Y260X/Y260X^* mice. Representative confocal images of base, middle, and apex cochlear turns of P14 *Tmprss3^Y260X/Y260X^*-treated mice immunostained against Myo7a (green) and phalloidin (blue). (**B**) Mean ± SD counts of IHCs and OHCs per 100-μm section from untreated (*n* = 4 ears) and treated P14 *Tmprss3^Y260X/Y260X^* (*n* = 7) mice measured in the base, middle, and apex cochlear turns. Each data point represents an individual cochlea. Nonparametric ANOVA with Tukey’s post hoc test for comparison of untreated versus treated IHC: *P* = 0.0003 for apex, *P* = 0.0008 for mid, *P* = 0.006 for base, and OHC: *P* = 0.009 for apex, *P* = 0.79 for mid, *P* = 0.78 for base.

Based on our results showing preservation of *Tmprss3^Y260X/Y260X^* HCs with in vivo reduction of EP in 2 separate 2-strain mouse models, we hypothesized that pharmacologic reduction of EP would also preserve HCs in *Tmprss3^Y260X/Y260X^* mice. Furosemide acts on the Na^+^-K^+^-2Cl^–^ cotransporter in the kidney to induce diuresis (29). Additionally, action of furosemide on the cotransporter in the cochlea leads to transient reduction in the EP (30). We tried varying amounts of furosemide injected intraperitoneally (50–200 mg/kg) between P10 and P14 and found that 200 mg/kg injected once daily was tolerated when saline was administered twice daily to counter expected diuresis. In a litter of 9 *Tmprss3^Y260X/Y260X^* mice, 2 served as controls and received daily administration of saline only, and 7 were injected with 200 mg/kg furosemide daily from P10 to P14. Animals treated with the furosemide had significantly increased IHC and OHC (in apex only) survival compared with saline-treated controls (IHC: *P* = 0.0003 for apex, *P* = 0.0008 for mid, *P* = 0.006 for base; OHC: *P* = 0.009 for apex, nonparametric ANOVA with Tukey’s post hoc test) (Figure 7). In contrast, administration of 100 mg/kg/d furosemide led to minimal or no HC preservation (*n* = 6; data not shown). These results suggest pharmacologic reduction in EP leads to preservation of cochlear HCs that lack functional TMPRSS3.

## Discussion

The EP is integral to cochlear physiology, with its functional significance becoming especially apparent during and after the onset of auditory function. In mature mice, EP typically stabilizes within the range of +80 to +100 mV. This stabilization is critical for sustaining the large electrochemical gradient necessary for the transduction process within HCs. Genetic defects that affect the formation or maintenance of EP are associated with hearing impairment in mice and humans. In this context, our findings introduce EP abnormalities as a contributing factor to the pathologic mechanism of DFNB8/10 HL.

Our prior observation that inner-ear organoids derived from *Tmprss3^Y260X/Y260X^* mice did not show HC degeneration indicated a possible extracellular mechanism for degeneration (24). This observation led us to explore the consequence of direct culturing of cochlear explants from control and *Tmprss3^Y260X/Y260X^* mice. We found that, in culture, we could prevent the rapid HC degeneration that occurs between P12 and P14 in *Tmprss3^Y260X/Y260X^* mice (Figure 3), further supporting our hypothesis that EP is an underlying contributor to the HC death.

To understand the directional effect and the magnitude of loss of Tmprss3 on EP, we measured EP from the cochleae of *Tmprss3^Y260X/Y260X^* and control mice. Surprisingly, we observed abnormally high EP levels in the maturing cochlea of *Tmprss3^Y260X/Y260X^* mice compared with controls (Figure 4). To date, many reported defects in EP that result in deafness are due to a reduction or failure to generate EP (31–33). Although the EP measured in the maturing cochlea of *Tmprss3^Y260X/Y260X^* mice was significantly elevated, it was not outside the physiologically normal range for mature cochlea (>P14). These findings indicate that maturing HCs may lack the resilience required to withstand premature elevations in EP, and part of the HC maturation process entails equipping the HCs to survive in environments characterized by high ionic gradients.

Next, we asked if reducing EP in vivo through genetic manipulation could preserve HCs in the *Tmprss3^Y260X/Y260X^* mice. EP is maintained by the stria vascularis, which is a specialized epithelial layer located on the lateral wall of the cochlear duct. This structure plays a critical role in regulating the ionic composition of the endolymph, the fluid that fills the scala media and bathes the HCs of the inner ear. The transcription factors *Pou3f4* and *Mitf* share critical roles in influencing the function of the stria vascularis, particularly through their effects on cellular development and integrity, which are essential for EP formation and maintenance. Mutant mouse models for both genes result in significant reduction of EP (26, 28). We crossed *Tmprss3^Y260X/Y260X^* mice with both the *Mitf^+/Mi-wh^* mutant and the *Pou3f4^delJ^* mutant mice. By eliminating the supraphysiologic rise in EP, we saw significant HC survival at the critical P12–P14 time point, and HC survival rates mimicked that of litter mate controls (Figure 5).

Complicating efforts to tease apart the mechanism(s) driving TMPRSS3-related HL is the lack of a specific, well-validated antibody to detect TMPRSS3 protein in mouse cochleae. However, *Tmprss3* RNA in mouse cochleae is expressed throughout the cells of organ of Corti, including IHCs, OHCs, and the spindle root cells, but no expression was detected within the stria vascularis and or in type II SGNs (7, 9). These data suggest TMPRSS3 functions within the sensory HCs of the cochlea.

To date, there are a limited number of potential downstream targets for TMPRSS3. Previously, the outward rectifying K^+^ channel, KCNMA1, was shown to be absent in the HCs of P12–P13 *Tmprss3^Y260X/Y260X^* mutants and has been hypothesized to be a downstream target of TMPRSS3. To test whether KCNMA1 was restored in surviving *Tmprss3^Y260X/Y260X^* HCs in vivo, we analyzed the KCNMA expression in double-mutant mice. Compared with controls (*Tmprss3^Y260X/+;^ Mitf^+/+^*), surviving HCs in the *Tmprss3^Y260X/Y260X^;Mitf^+/Mi-wh^* model also did not show expression KCNMA1 from P12 to P15, (Figure 6), indicating that KCNMA1 expression in surviving HCs requires TMPRSS3. It should also be noted that KCNMA1 mutant mice are not profoundly deaf and develop a late-onset, high-frequency, mildly progressive HL (16). The role KCNMA1 loss plays in the pathobiology of TMPRSS3-related HL remains unclear.

*Pou3f4^delJ^* mice and *Mitf^+/Mi-wh^* mice are deaf due to a failure to generate EP, and both lines have progressive HC loss. Although the decrease in EP resulted in significant HC survival for TMPRSS3 mutants crossed with either line, ultimately these crosses are still deaf. To circumvent the effects of genetic manipulation, we sought to regulate EP pharmacologically by manipulating electrolyte balance. Using furosemide, a loop diuretic, we treated *Tmprss3^Y260X/Y260X^* mice with varying dosing regimens. When *Tmprss3^Y260X/Y260X^* mice were treated systemically with this drug, we saw an increase in HC survival (Figure 7), during the critical P12–P14 time point, primarily in the apex. HC survival was not as robust as what we saw with complete oblation of EP via genetic manipulation (Figure 5). It is unclear to what extent and duration furosemide lowered EP in treated animals, and further studies are needed to explore dosing and its impact on regulating EP. Nevertheless, loop diuretics may provide a mechanism to extend the therapeutic window for *TMPRSS3* HL in humans. This is particularly important given that humans develop hearing, and EP, in utero and may be born with permanent HC loss. Pharmacologic reduction of EP may provide a method for preservation of cochlear HCs to allow for later gene therapy intervention (29).

In humans, pathogenic variants in *TMPRSS3* result in 2 distinct phenotypes: congenital severe-to-profound HL (DFNB10) and postlingual progressive HL (DFNB8). We suspect the DFNB10 phenotype is associated with a toxic increase in EP that results in HC death and profound HL. Whereas the pathomechanism for DFNB8 remains elusive, it is less likely tied to EP, given its milder phenotype. More work is needed to understand the full extent of TMPRSS3’s role in auditory function.

In summary, we implicate defects in EP as the possible pathomechanism underlying severe-profound *TMPRSS3*-related HL. We show through multiple lines of evidence that HL in *Tmprss3^Y260X/Y260X^* mice is due to a toxic premature increase in EP that results in subsequent HC death just before and at the onset of hearing. We also show, by manipulating EP, either by genetic manipulation or pharmacologically, HC death can be eliminated or diminished, indicating a mechanism for treatment of *TMPRSS3*-related HL.

## Methods

### Sex as a biological variable.

Our study examined male and female animals, and similar findings are reported for both sexes.

### Data availability.

Further information and requests for resources and reagents should be directed to and will be fulfilled by the corresponding author. Research reagents generated in this study will be distributed upon request to other investigators under a material transfer agreement. Microscopy data reported in this article will be shared by the corresponding author upon request. Original code is not reported in this article. Any additional information required to reanalyze the data reported in this work is available from the corresponding author upon request.

### Experimental model details.

The mice were housed in a temperature-controlled room with a 12-hour light/dark cycle and had free access to water and food. Male and female mice were used in all experiments (P7–P48). *Tmprss3^Y260X/Y260X^* mice (C3HeB/FeJ background) were provided by Michel Guipponiat (University of Geneva, Geneva, Switzerland). C3HeB/FeJ (RRID: IMSR_JAX:000658), C3HeB/FeJ-*Pou3f4^del-J^*/J (RRID: IMSR_JAX:004406), and B6.Cg-*Mitf^Mi-wh^*/J (RRID: IMSR_JAX:000057) mice were purchased from The Jackson Laboratory. *Tmprss3^Y260X/Y260X^*;*Pou3f4^del-J^* double-mutation mice were generated by crossbreeding *Tmprss3^Y260X/Y260X^* and C3HeB/FeJ-*Pou3f4^del-J^*/J. *Tmprss3^Y260X/Y260X;^Mitf^Mi-wh^*/J hybrid strain mice were generated from backcrossing B6.Cg-*Mitf^Mi-wh^*/J to C3HeB/FeJ to obtain C3.Cg-*Mitf^Mi-wh^* followed by crossbreeding *Tmprss3^Y260X/Y260X^* and C3.Cg-*Mitf^Mi-wh^*. The *Mitf^+/Mi-wh^* mouse recapitulates human Waardenburg syndrome in that the mutation functions in a dominant-negative fashion. Heterozygous *Mitf^+/Mi-wh^* mice have profound HL, lack of EP, and altered fur coloring. *Mitf^Mi-wh/Mi-wh^* share these same characteristics but are less viable and poor breeders. For these reasons, we used *Tmprss3^Y260X/Y260X;^Mitf^+/Mi-wh^* as our experimental animals.

### Genotyping.

Genomic DNA was prepared from the mouse ear notch with QuickExtract DNA extraction solution, following the manufacturer’s instructions. For the *Tmprss3^Y260X/Y260X^* mice, PCR amplicons were generated using 10 pmol of each primer (TMP3SEQ_F and TMP3SEQ_R) and then sequenced with TMP3SEQ_F primer to determine the genotype of the mice. For *Pou3f4^del-J^* mice, the TaqMan quantitative PCR protocol (protocol 41591 from The Jackson Laboratory) was performed on QuantStudio 5, and the comparative Ct values of *Pou3f4* and housekeeping *Apob* gene were calculated. The ratio of Ct values of *Pou3f4* mice were compared to Ct values of confirmed wild-type, hemizygous, and homozygous *Pou3f4* mice to determine the genotype of unknown sample. For *Mitf^+/Mi-wh^* mice, direct sequencing was performed using a commercial laboratory (TransNetyx). The list of all primers and sequences used for genotyping can be found in Supplemental Table 1.

### Scanning electron microscopy.

The P14 mice were euthanized by decapitation. The cochlea was removed from the temporal bone and fixed for 24 hours with 2.5% glutaraldehyde in 0.1 M sodium cacodylate buffer (pH 7.4) containing 2 mM CaCl_2_, washed in buffer. The sensory epithelium was harvested by removing the bony cartilage, the lateral wall, Reissner’s membrane, tectorial membrane, and the cochlear nervous tissue under the stereomicroscope and was post-fixed for 1 hour with 1% OsO_4_ in 0.1 M sodium cacodylate buffer and washed. The tissues were dehydrated via an ethanol series, critical point dried from CO_2_, and sputter coated with gold. The morphology of the HCs was examined in an FEI Quanta 200 scanning electron microscope (Thermo Fisher Scientific) and photographed.

### FM 1-43FX staining.

The P7 pups were euthanized by decapitation. The cochlea was removed from the temporal bone and placed in ice-cold DMEM/F12 medium. The sensory epithelium was harvested by removing the bony cartilage, the lateral wall, Reissner’s membrane, tectorial membrane, and the cochlear nervous tissue under the stereomicroscope prior to FM 1-43FX staining. The 10 mM stock solution of FM 1-43FX was prepared in DMEM/F12. The sensory epithelium was stained with 10 mM FM 1-43FX for 30 seconds at room temperature (RT) and immediately washed with DMEM/F12. The tissue was transferred to the glass slide within DMEM/F12, covered with coverslips, visualized on Leica DMi8 microscopy equipped with epifluorescence optics and Leica Y3 filter cube (excitation 545 nm, emission 605 nm).

### SGN subtypes.

The P11 and P21 pups were euthanized by decapitation. The cochlea was removed from the temporal bone and fixed with 4% paraformaldehyde overnight at 4°C. Cochlea were then decalcified in 120 mM EDTA for 1 day (P11) or 3 days (P21) at RT. The EDTA was refreshed daily, and the elasticity of bone structure was checked to confirm the complete decalcification. The cochlea was cryoprotected in 30% sucrose in PBS and embedded in TF Tissue Freezing Medium. Modiolar sections of 6 μm were cut on a cryostat and air-dried for 2 hours. The sections were permeabilized with 0.5% Triton X-100 in PBS for 10 minutes at RT, blocked with 5% goat serum in PBS overnight at 4°C, and incubated with primary antibody (tubulin β-3 for type I, calretinin for type IA, calbindin for type IB, Brn-3a for type IC, and NGFR for type II SGNs) in 5% goat serum in PBS overnight at 4°C. After washing with 0.01% Trixon X-100 in PBS 3 times for 10 minutes each, the sections were incubated with secondary antibodies (Alexa Fluor 488 conjugated goat anti–rabbit IgG; Alexa Fluor 647 conjugated goat anti–mouse IgG1; Alexa Fluor 568 conjugated goat anti–mouse IgG2a; and Alexa Fluor 647 conjugated goat anti–rabbit IgG) in 5% goat serum in PBS for 1 hour at RT. The sections were washed and mounted with ProLong Gold Antifade Mountant with DAPI. Staining was visualized on Leica DMi8 microscopy equipped with epifluorescence optics. The percentages of SGN subtypes were quantified by calculating the number of immunopositive cells for each marker relative to the total number of SGNs (Figure 2). The total number of type I SGNs was determined by counting TUJ1-positive cells within a 10,000 μm³ volume, defined as a 100 μm × 100 μm area with a depth of 1 μm (Supplemental Figure 1). Details of all antibodies used, including the source and catalog number, are included in Supplemental Table 1.

### Mouse cochlea explant culture.

The explant cultures were performed on both P14- and P35-equivalent explants. For P14-equivalent explants, pups from *Tmprss3^Y260X/Y260X^* and *Tmprss3*^+/–^ control littermates were euthanized by decapitation at P7. The sensory epithelium was harvested by removing the bony cartilage, the lateral wall, Reissner’s membrane, tectorial membrane, and the cochlear nervous tissue under the stereomicroscope. The sensory epithelium was transferred to 6-well Millicell Cell Culture Inserts with a Pasteur pipette and cultured for 7 days at 37°C in DMEM/F12 medium supplemented with 100 mg/mL Normocin. For P35-equivalent cochlear explants, cochleae were harvested from *Tmprss3^Y260X/Y260X^* animals and *Tmprss3*^+/–^ control littermates at P5, plated on glass cover slips, and cultured in 1% FBS, HEPES, and MEM + GlutaMax for up to 30 days (D30; the equivalent of P35). The sensory epithelium was then fixed with 4% PFA for 20 minutes at RT and stained for Myosin7a (Myo7a). Details of all antibodies used, including the source and catalog number, are included in Supplemental Table 1.

### Physiologic auditory system evaluation in mice.

Auditory evaluation using ABR and otoacoustic emissions were performed as described previously (34).

### EP measurement.

For P7–P24 mice, measurement of EP was performed while mice were fully anesthetized using ketamine/xylazine and immobilized in the ventral position. The head was further immobilized and stabilized using a suture passed around the maxillary central incisors. Sterile technique was used throughout the procedure. We first began with a midline incision to expose the trachea. After dividing the strap muscles, a simple tracheotomy was performed to allow CO_2_ egress during immobilization, which can lead to reduction in EP (12). The left bulla was then identified and dissected, allowing exposure of the cochlea. A 1 mm diamond bit on a high-speed drill was used to remove the bone just above the basal turn lateral to the round window membrane. Care was taken to not disrupt the membranous labyrinth. A pulled-glass capillary microelectrode (3–5 MΩ) was filled with 150 mM KCl and mounted on a micromanipulator. The ground electrode was placed into neck soft tissue. The microelectrode was then advanced into the scala media through the stria vascularis while measuring the response. Voltage was recorded before advancing into the scala media and 0 mV was set as the reference. The DC voltage was amplified (AxoPatch 200B Patch Clamp Amplifier; Molecular Devices) ×10 and continuously acquired using pCLAMP (Axoscope 11). An appropriate potential was identified when a measurement was stable for at least 5 seconds and the potential disappeared with advancement of the microelectrode into the scala vestibuli.

For P28 mice, measurement of EP was performed as previously described (12). Briefly, P28 mice were anesthetized with 2,2,2-tribromoethanol (T4842; Sigma-Aldrich) at a dose of 0.35 mg/g body weight. EP measurements were made using glass microelectrodes inserted into the round window and through the basilar membrane of the first turn of the cochlea. Data were recorded digitally (Digidata 1440A and AxoScope 10; Axon Instruments) and analyzed using Clampfit10 (RRID: SCR_011323; Molecular Devices). Six *Tmprss3^Y260X/Y260X^* and 6 control mice (equal ratios of male and female mice) were evaluated. All members of the research team were blinded to the genotype of the mice during EP testing. The 2 groups of mice were handled similarly to control for confounding variables (e.g., cage location, feeding time).

### Whole-mount immunofluorescence.

The pups between P16 and P31 were euthanized by decapitation. The cochlea was then removed from the temporal bone, then decalcified in 120 mM EDTA for 3 to 4 days at RT. EDTA was refreshed daily with sponginess checked to confirm complete decalcification. The sensory epithelium was harvested by removing the bony cartilage, the lateral wall, Reissner’s membrane, tectorial membrane, and the cochlear nervous tissue under the stereomicroscope prior to permeabilization by 1× PBS/0.5% TritonX-100 at RT. Tissue was blocked overnight in 5% goat serum/1× PBS in 4°C and incubated with primary antibody (Myo7a for HCs) in 5% goat serum in PBS overnight at 4°C. After washing with 0.01% Trixon X-100 in PBS 3 times for 10 minutes each, the sections were incubated with secondary antibodies (Alexa Fluor 568 conjugated goat anti–rabbit IgG; Alexa Fluor 555 conjugated goat anti–rabbit IgG; and Alexa Fluor 488 conjugated goat anti–mouse IgG2a) in 5% goat serum in PBS for 1 hour at RT. Tissue was washed again 3 times in 1× PBS/0.01% TritonX-100 for 10 minutes at RT. Hoechst 33342 in 1× PBS was added after the last wash. Tissue was briefly rinsed with 1× PBS and transferred onto a slide. Using a stereoscope, the tissue was oriented so that the HCs were face up, mounted with Invitrogen ProLong Gold Antifade Mountant with DAPI, and allowed to dry overnight. Slides were visualized using a Zeiss LSM 880 confocal microscope. HC count analysis was performed manually using ImageJ (NIH). Details of all antibodies used, including the source and catalog number, are included in Supplemental Table 1.

### KCNMA1 quantification.

For KCNMA1 quantification, blocking was performed for 1 hour in 2.5% normal donkey serum and stained at 4°C overnight with primary antibodies (mouse anti-Myo7a, mouse anti-parvalbumin, and rabbit KCNMA1/KCa 1.1). After washing with PBS, samples were incubated with secondary antibody (Alexa Fluor 488 conjugated goat anti–mouse IgG2a and Alexa Fluor 555 conjugated goat anti–rabbit IgG) and Alexa Fluor Plus 405 Phalloidin. Confocal imaging was performed using 10× air and 63× oil-immersion objectives with a Zeiss LSM 800 confocal microscope. Maximum intensity projection images were generated in Image J. KCNMA1 punctae were counted using automated quantification with Image J.

### Pharmacologic reduction of EP using furosemide.

Mice were injected intraperitoneally with varying amounts of furosemide diluted in saline from P10 to P14 from 50 mg/kg to 200 mg/kg. A dose of 200 mg/kg daily with twice-daily saline boluses (100 μL/g) has been shown to be well tolerated and to effectively reduce the EP in mice (35).

### Statistics.

Data are presented as mean ± SD and were derived from at least 3 independent experiments, unless otherwise indicated. Statistical analysis was carried out in GraphPad Prism 9, SAS version 9.4 (SAS Institute), and R software (R Foundation for Statistical Computing, http://www.r-project.org/). The exact Wilcoxon rank-sum test (exact Mann-Whitney *U* test) was used to compare groups for data that were not normally distributed and an unpaired *t* test was used to compare group means for normally distributed data. The nonparametric ANOVA with Tukey’s post hoc test (36, 37) was used to compare untreated and treated mice in base, middle, and apex cochlear turns. All statistical tests were 2-sided, with statistical significance set at α = 0.01.

### Study approval.

All animal experiments were approved and performed in compliance with the guidelines provided by the IACUC at Indiana University School of Medicine (protocol 23148) or at Boston Children’s Hospital (protocol 000013339).

### Data availability.

Values underlying graphed data and reported means presented in both the main text and supplemental material are included in the Supporting Data Values file.

## Author contributions

AES, JRH, and RFN conceptualized the study. AES, YSC, SLR, XW, JMF, KTAB, JM, NBL, JL, HYK, MH, RO, JYL, EC, DJT, BZ, JRH, and RFN contributed to the methodology and the investigation. AES, RJH, and RFN contributed resources, contributed to visualization of data, and supervised the work. AES and RFN wrote the original draft of the manuscript, and AES, MH, KTAB, JRH, and RFN reviewed and edited the manuscript. AES, DJT, MH, JRH, BZ, and RFN contributed to funding acquisition.

## Supplementary Material

Supplemental data

Supporting data values

## Version 1 07/17/2025

In-Press Preview

## Version 2 09/16/2025

Electronic publication
