# Supplementary material for: Endocochlear potential contributes to hair cell death in TMPRSS3 hearing loss
Source: J Clin Invest. 2025 Jul 17;135(18):e186395. doi: 10.1172/JCI186395 (PMC12435832; doi:10.1172/JCI186395)
Supplement: Supplemental data [file jci-135-186395-s337.pdf]

Endocochlear potential contributes to hair cell death in TMPRSS3 hearing loss

Supplemental information

Figure S1

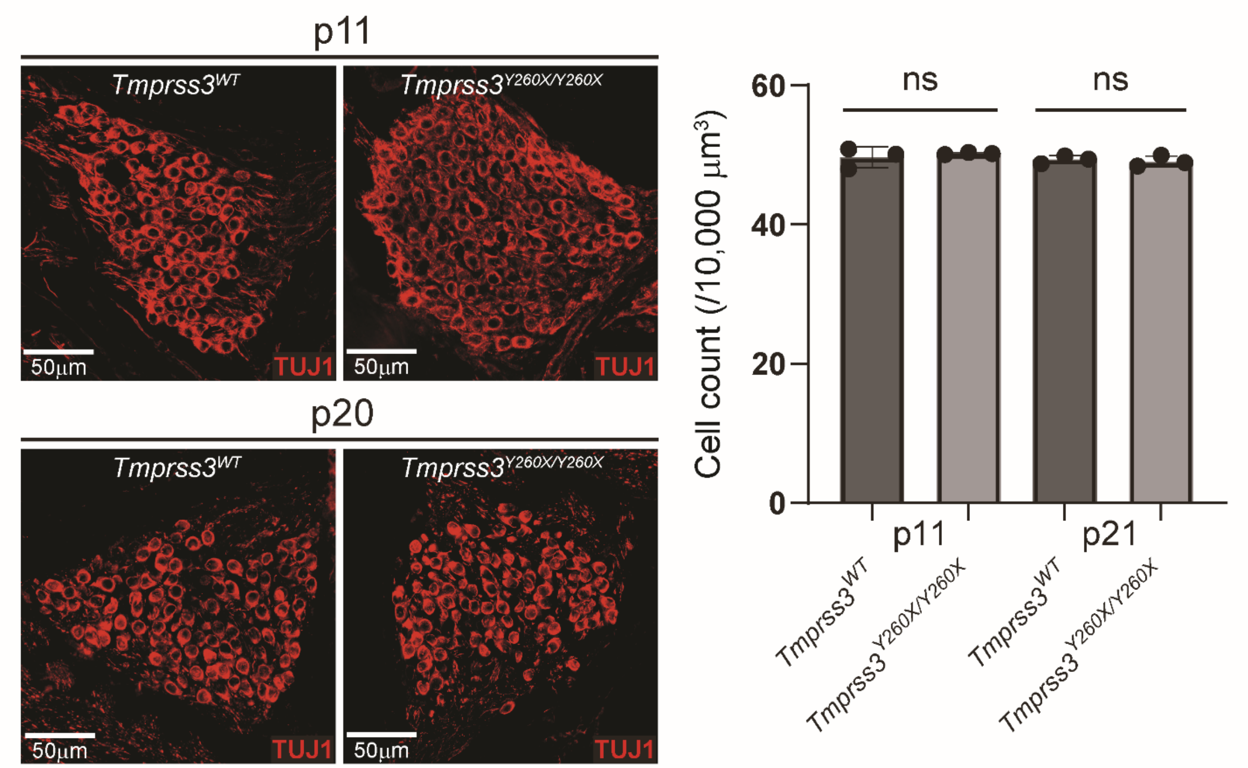

Supplemental Figure S1: Spiral ganglion neuron counts (TUJ1$^+$ neurons) are not significantly different between *Tmprss3*$^{Y260X/Y260X}$ mice and controls (WT) at postnatal day 11 (p11) and p21.

Figure S2

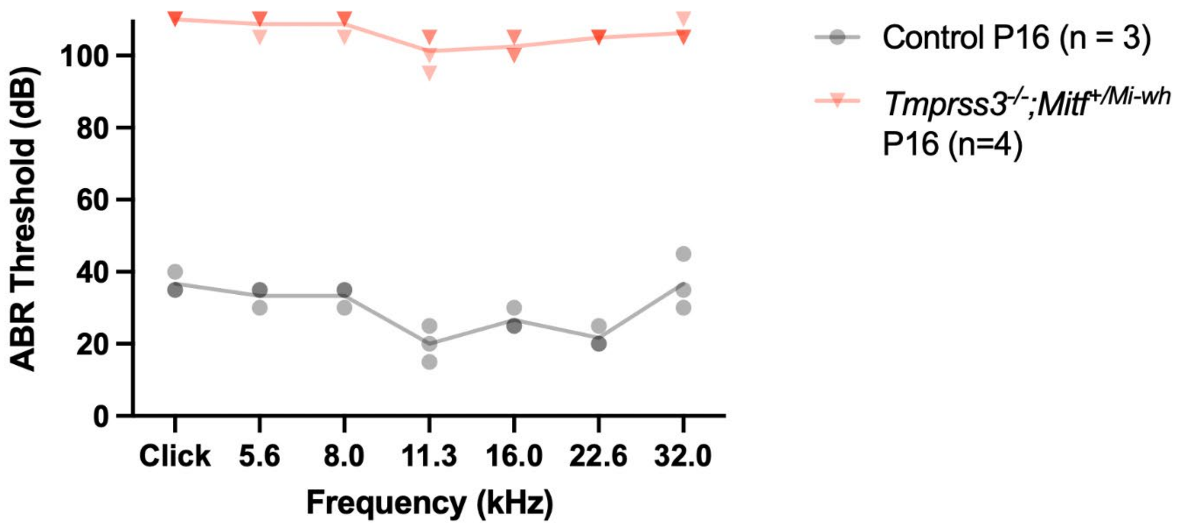

Supplemental Figure S2. Two-strain cross of *Tmprss3*$^{-/-}$ with *Mitf*$^{+/Mi\text{-}wh}$ rescues hair cells but does not restore hearing. ABRs from P16 mice show no recovery of hearing in the setting of preserved inner and outer hair cells (Figure 5).

Figure S3

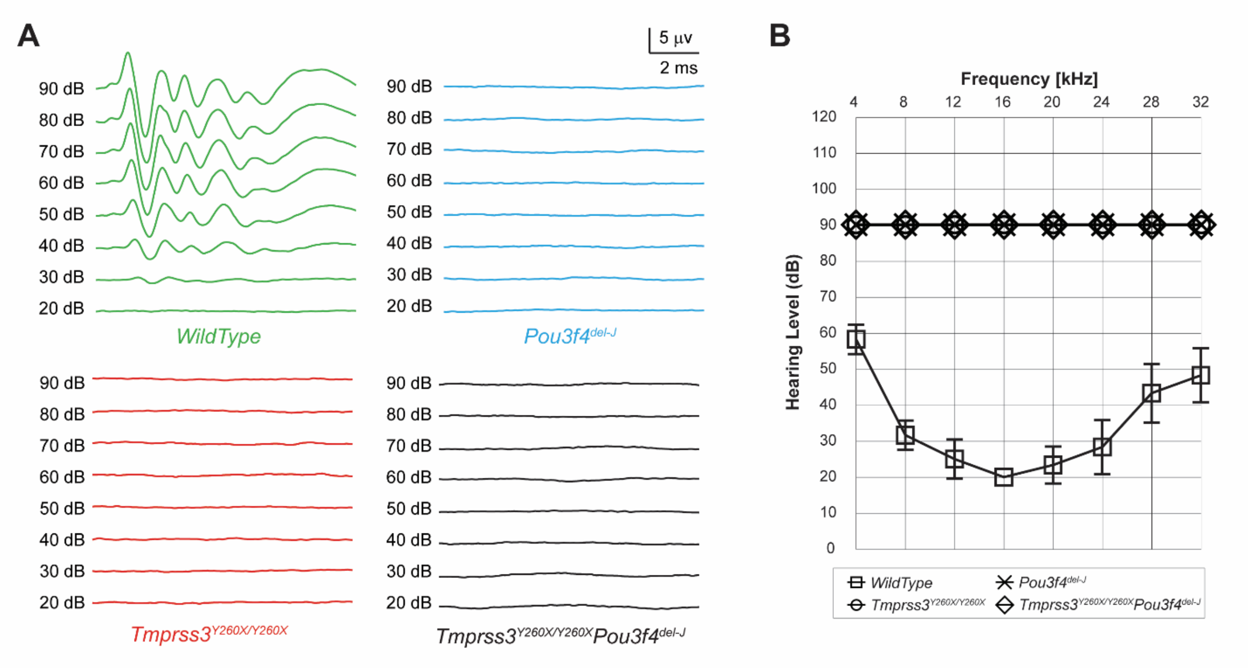

Supplemental Figure S3. Two-strain cross of *Tmprss3*$^{Y260X/Y260X}$ with *Pou3f4*^{delJ} rescues hair cells but does not restore hearing. (**A**) Representative ABR tracings from control (Wildtype), individual strain and double mutant mice at P28. (**B**) Average pure tone averages for Wildtype (n = 6), *Pou3f4*^{delJ} (n = 8), *Tmprss3*$^{Y260X/Y260X}$ (n = 6), and *Tmprss3*$^{Y260X/Y260X}$;*Pou3f4*^{delJ} (n = 6) strains at P28 show no recovery of hearing in both male and female mice in the setting of preserved inner and outer hair cells (Figure 5).

Supplemental Table 1

Key resources table

REAGENT or RESOURCE	SOURCE	IDENTIFIER
Antibodies		
Rabbit anti-Myosin-VIIa (1:200)	Proteus Biosciences	Cat# 25-6790; RRID:AB_10015251
Mouse anti-Myosin-VIIa (1:200)	Santa Cruz Biotechnology	Cat# SC-74516; RRID:AB_2148626
Mouse anti-Sox2 (1:100)	BD Biosciences	Cat# 561469; RRID:AB_10694256
Mouse anti-Tubulin Beta 3 (1:300)	BioLegend	Cat# 801202 RRID:AB_2313773
Rabbit anti-Brn-3a (1:100)	Millipore	Cat# AB5945; RRID:AB_92154
Rabbit anti Calbindin D-28k (1:700)	Swant	Cat# CB-38a
Mouse anti-Calretinin (1:700)	Millipore	Cat# MAB1568; RRID:AB_94259
Rabbit anti-NGFR (1:1900)	Cell Signaling	Cat# 8238S
Mouse anti-Parvalbumin (1:250)	Sigma-Aldrich	Cat# P3088; RRID:AB_477329
Rabbit anti-KCNMA1/KCa 1.1 (1:500)	Alomone Labs	Cat# APC-021; RRID:AB_2313725
Alexa Fluor™ 568 Phalloidin	Thermo fisher	Cat# A12380
Alexa Fluor™ Plus 405 Phalloidin	Thermo fisher	Cat# A30104
FM1-43FX (fixable version of FM1-43)	Thermo fisher	Cat #F35355
Goat anti-Rabbit IgG (H+L) Cross-Adsorbed Secondary Antibody, Alexa Fluor™ 488	Thermo fisher	Cat# A11008 RRID: AB_143165
Goat anti-Mouse IgG1 Cross-Adsorbed Secondary Antibody, Alexa Fluor™ 647	Thermo fisher	Cat# A21240 RRID: AB_2535809
Goat anti-Mouse IgG2a Cross-Adsorbed Secondary Antibody, Alexa Fluor™ 568	Thermo fisher	Cat# A21134 RRID: AB_2535773
Goat anti-Rabbit IgG (H+L) Cross-Adsorbed Secondary Antibody, Alexa Fluor™ 647	Thermo fisher	Cat# A21244 RRID: AB_2535812
Goat anti-Rabbit IgG (H+L) Highly Cross-Adsorbed Secondary Antibody, Alexa Fluor™ 568	Thermo fisher	Cat# A11036 RRID: AB_10563566
Alexa Fluor™ goat anti-rabbit IgG 555	Thermo fisher	Cat# A27039 RRID: AB_2536100
Alexa Fluor™ goat anti-mouse IgG2a 488	Thermo fisher	Cat# A21131 RRID: AB_2535771
Chemicals, peptides, and recombinant proteins		
Lucigen Corporation QuickExtract DNA Extraction Solution 1.0, 50 mL	Fisher Scientific	Cat# NC9904870
TaqMan™ Fast Advanced Master Mix	Thermo Scientific™	Cat# 4444557
Invitrogen™ PCR SuperMix	Thermo Scientific™	Cat# 10-572-014
Invitrogen™ ProLong™ Gold Antifade Mountant with DNA Stain DAPI	Fisher Scientific	Cat# P36931

Oligonucleotides		
TMP3SEQ_F<br>5'-*CCCGAGATTTGGCAGTATTG*-3'	Fasquelle et al.	N/A
TMP3SEQ_R<br>5'-*AGCAGGCCCAGTCACTCAC*-3'	Fasquelle et al.	N/A
Pou3f4$^{del\text{-}J}$ mice genotyping primer 58714 mutant F:<br>5'-*CAACCCCTCAATGTGTACTCG*-3'	Jackson lab Protocol 41591	N/A
Pou3f4$^{del\text{-}J}$ mice genotyping primer 58715 mutant R:<br>5'-*ATGCAGGCTCTGTGTGGAG*-3'	Jackson lab Protocol 41591	N/A
Pou3f4$^{del\text{-}J}$ mice genotyping primer 58716 mutant Probe:<br>5'-*CCAGGCTTCACCGTGAGCGGTAT*-3'	Jackson lab Protocol 41591	N/A
Pou3f4$^{del\text{-}J}$ mice genotyping primer oIMR1544 Apob F:<br>5'-*CACGTGGGCTCCAGCATT*-3'	Jackson lab Protocol 41591	N/A
Pou3f4$^{del\text{-}J}$ mice genotyping primer oIMR3580 Apob R:<br>5'-*TCACCAGTCATTTCTGCCTTTG*-3'	Jackson lab Protocol 41591	N/A
Pou3f4$^{del\text{-}J}$ mice genotyping primer TmoIMR0105 Apob Probe:<br>5'-*CCAATGGTCGGGCACTGCTCAA*-3'	Jackson lab Protocol 41591	N/A
